# News in Brief

**Published:** 1922-02

**Authors:** 


### NEWS IN BRIEF.

### What the Provinces are Doing—Personal Notes—Christmas Festivities.

Mr. W. H. S. Oulton, LL.M., has been elected chairman of the David Lewis Northern Hospital, Liverpool. He joined the committee in 1908, and became chairman of the weekly board in 1914.

The Local Committee for Exeter and Devon, under the Voluntary Hospitals' Commission, has appointed an honorary secretary, and approved a grant of £200 a year towards his travelling and out-of-pocket expenses. The Committee suggests that the County Council should pay 5/6ths and the City Council 1/6th of this sum.

Though started only in 1918, the East Lancashire Workpeople's Hospital Fund now averages £10,000 a year, for the benefit of the Blackburn Royal Infirmary and other charities. The founder of the Fund, Mr. Lawrence Cotton, J.P., died last year. It is now hoped to raise sectional subscriptions from all classes.

Mr. H. B. Harper, secretary of the Alton Cripples Hospital, has been elected a member of the East Ham Borough Council, In this, his first election, Mr. Harper defeated the sitting Labour member.

The appointment of Dr. R. H. A. Plimmer to the Chair of Medical Chemistry at St. Thomas's Hospital Medical School in the University of London has led to his resignation of the headship of the bio-chemical department of the Rowett Institute (Craibstone) of Research in Animal Nutrition, Aberdeen.

The Maternity Hospital, New Bridge Street, Newcastle, is contemplating removal to the premises occupied by the Industrial Schools in City Road. The Schools will move in March to Axwell Park ; and if the Corporation will purchase the Schools' site for the hospital, the hospital's present valuable site might be given in exchange to the Corporation.

The South Devon and East Cornwall Hospital is expected to become the medical school of the proposed University of the South-West.

The King has permitted the Great Northern Central Hospital to assume the style of Royal. It will, therefore, now be known as the Royal Northern Hospital.

The Nottingham Hospital Saturday Fund has nearly doubled its previous total, having raised £28,682, compared with £15,843 last year.

Lieut.-Col. Percy Gotch Robinson has been asked to act as President of the Bristol Royal Infirmary pending the formal election in a few months' time. A keen sportsman, he was once captain of the Clifton College cricket eleven.

Mr. J. G. Ashley has been appointed honorary secretary and treasurer of the Teddington and Hampton Wick Cottage Hospital in succession to Mr. F. H. Munly.

The South London Hospital for Women has started a Pound for Pound Fund to raise the equivalent of the £800 emergency grant from the Voluntary Hospitals Commission.

The marriage of a medical peer is announced with the engagement of Lord Cobham to Mrs. Gershoi, of Hove. The son of the late Dr. R. G. Alexander, senior physician to the Halifax Royal Infirmary, Lord Cobham was himself in the Mental Hospital Medical Service until the barony was formally called out of abeyance shortly after his father's death in 1916.

The Sheffield Royal Infirmary and the Sheffield Royal Hospital decided to introduce patients' payments on January 1 last. This has led each hospital to appoint a lady almoner at a salary of £150 a year, with meals.

Eventually, the General Hospital, Birmingham, has decided that all patients, subject to a few exceptions, be required to pay a sum of two guineas a week toward the cost of their maintenance, according to their means. This step has been taken as a last resort, since local effort has not in the meantime reduced the hospital's liabilities.

The hospital for children suffering from surgical tuberculosis at Gringley-on-the-Hill, Notts, which the Duke of Portland and General Sir Joseph Lacock have maintained for the past ten years, is threatened with closing. Its supporters are unable to finance it, but appealing to the local authorities to maintain it, they offer to subscribe £200 a year each.

Officers of twelve hospitals in Birmingham and Dudley have presented an illuminated address to Mr. G. C. Simnett, who has been hon. secretary of the Blackheath and Rowley Hospital Committee for 21 years.

Mr. J. T. Holmes, honorary treasurer to the York Workpeople's Hospital Fund, has resigned to take an appointment at St. Anne's-on-Sea. He has held the post for eight years, during which the fund has advanced considerably. Miss F. H. Maw is the new treasurer.

Patients from 182 towns, including Manxmen, seek treatment at the Manchester Ear Hospital, which is therefore seeking support from places as distant as Hereford.

The appointment of Lord Stanmore to the treasurership of St. Bartholomew's Hospital fills the vacancy created by the death of Viscount Sandhurst. Educated at Winchester and Trinity College, Cambridge, the new treasurer succeeded in 1912 to the peerage conferred on his father, Sir Arthur Hamilton-Gordon, a Colonial Governor. In 1914 Lord Stanmore became a Lord-in-Waiting to the King. He had previously contested North Dorset in the Liberal interest.

Aldingbourne House, near Chichester, has been purchased by the West Sussex County Council for a sanatorium. It contains, including 15 shelters, 59 beds. Of the 120 acres, 35 are reserved for the institution, 85 for small holders. The net cost to the county will be £500 per bed. The institution will treat ex-Service men suffering from phthisis, and patients unable to pay the three guineas a week charged at the Midhurst Sanatorium.

At the annual dinner of the Incorporated Society of Hospital Officers, when Viscount Hambledon was the guest of the evening, Sir James Michelli, who presided, reiterated his plea for a pension scheme for hospital officers on a non-contributory basis.

At Walthamstow the fire engine was requisitioned to convey appropriate gifts—and, incidentally, Councillor Warrington as Santa Claus—to the little inmates of the Council's Isolation Hospitals at "Brookfield," Hale End, and at Chingford. At the entrance to "Brookfield" a donkey was laden with the Christmas presents, and led into the grounds, much to the delight of the youngsters. These proceedings were filmed and appeared at the local picture theatre.

At St. George's Hospital, in addition to the usual Christmas festivities, there was a Christmas tree in the children's ward, and a special children's party in the Out-Patient Department, with an entertainment organised by the nurses. On January 3 the nurses had an evening party with private theatricals arranged amongst themselves. The fund for providing the Christmas gifts, decorations and dainties was very kindly contributed by residents in the neighbourhood and the lady visitors, so that no part of the cost of the festivities fell on the general funds of the hospital.

At the City of London (Chest) Hospital a feature of the Christmas celebrations was the singing of carols by the choirboys of St. Simon Zelotes and the Choral Society of St. James the Great. Everything was done to make the patients happy, the resident doctors and nursing staff cordially entering into the arrangements. Nor did the festivities end with Christmas, for the nursing staff arranged another entertainment for the patients, and on January 7 a pantomime was given by amateur performers.

The London Homœopathic Hospital, Great Ormond Street, held its annual Christmas-tree festivity on December 28, when patients and friends were equally affected by the spirit of good cheer which prevailed. Father Christmas, amid much excitement, distributed the toys with which the tree was laden. Miss Peggy O'Neill, from the Savoy Theatre, played the part of fairy godmother, bringing a large number of dolls to delight the little ones.

Sir Alfred Mond, Minister of Health, has promised to speak at the Central Poor Law Conference at the Guildhall on February 14, which will be opened by the Lord Mayor, and will be attended by delegates from all the Poor Law districts of England and Wales. The papers to be read are, on February 14, on the treatment of distress arising from unemployment, by Mr. C. H. Leach, Clerk of the Darlington Board of Guardians ; and, on February 15, on the use of Poor Law hospitals and the training of nurses, by Mrs. Eustace H. Lipscomb (St. Albans).

